# Is It Possible to Predict the Concentration of Natural Volatile Organic Compounds in Forest Atmosphere?

**DOI:** 10.3390/ijerph17217875

**Published:** 2020-10-27

**Authors:** Geonwoo Kim, Sujin Park, Dooahn Kwak

**Affiliations:** 1 Forest Welfare Division, Forest Policy and Economics Department, National Institute of Forest Science, Seoul 02455, Korea; bkim5020@korea.kr (G.K.); dkwak@korea.kr (D.K.); 2 Department of Environmental Health Sciences, Graduate School of Public Health, Seoul National University, Seoul 08826, Korea

**Keywords:** natural VOC, forest therapy, aroma therapy, betula platyphylla, VOC modeling

## Abstract

We aimed to understand the correlation between the microclimate environment within a forest and NVOC (Natural volatile organic compounds) concentration and the concentration of NVOC more efficiently through the prediction model method. In this study, 380 samples were collected and analyzed to examine the characteristics of NVOC emitted from a birch forest. NVOC were analyzed in May and July 2019, and measurements were performed at three different locations. Using a pump and stainless-steel tube filled with Tenax-TA, 9 L of NVOC was collected at a speed of 150 mL/h. The analysis of NVOC composition in the forest showed that it comprised α-pinene 27% and camphor 10%. Evaluation of the correlation between the NVOC concentration and the microclimate in the forests showed that the concentration increased markedly with the increase in temperature and humidity, and the concentration decreased with the increase in wind velocity. Nineteen substances in total including α-pinene and β-pinene were detected at high concentrations during the sunset. The results of the study site analysis presented a significant regression model with a R^2^ as high as 60.1%, confirming that the regression model of the concentration prediction of NVOC in birch forest has significant explanatory power.

## 1. Introduction

People have comfortable lives with industrialization and urbanization, but paradoxically are more exposed to many stressful environments as their urban lives become more advanced. Exposure to stressful environments such as noise, fear of accidents, effects of environmental pollution, and complex lifestyle in urban areas can affect an individual’s mental and physical health and may cause social problems [1,2]. In a survey conducted by the Korea Centers for Disease Control and Prevention (8991 Korean citizens over 19 years old), 29.1% (men: 26.2%, women: 32.0%) of the respondents considered their stress levels to be either ‘high’ or ‘very high’ [3]. Air pollution is one of the reasons for causing stress in the urban environment and chronic diseases, which contains anthropogenic volatile organic compounds (AVOC) as its main factors. The existence of AVOC within the big cities’ atmosphere environment have been emitted through vehicles, architectural materials, and industrial facilities. Some of them in itself present either harmful toxic or carcinogenesis and mutation traits for health [4].

Values regarding achieving quality of life by escaping from these stressful environments are becoming within the social life, which also aims for health and happiness. The pursuit of physical comfort is instinctive in most human beings; therefore, the influence of the natural environment on humans is significant. From the human perspective, the natural environment can be defined as a forest that restores the balance and harmony of the human body and provides comfort and restfulness [5,6]. Terms like healing forest and forest medicine [7] are associated with forests in China, Japan, and Korea. In countries that consider a forest environment as a means of healing, there is a growing social demand to promote health and well-being by utilizing forests as a therapeutic space [8,9,10]. In the case of Korea, the government designated healing forests by passing the Forestry Culture and Recreation Act and established a valuation basis and legal definition of healing forests. Natural volatile organic compounds (NVOC), representing the healing forest factor, are widely known as ‘phytoncides’ in Korea. Several studies have shown that NVOC emitted from forests have various noticeable therapeutic effects such as disease prevention and health improvement. Forest environment encouraged activity of human natural killer (NK) cells, percentage of NK cells, and intracellular anti-cancer proteins in lymphocytes [11], and this increased NK activity lasted for more than a week in both male and female subjects after several stays in forests [12]. Some studies have reported on physical changes like stabilizing blood-pressure, decrease in stress, and improving from tense to relaxed through exposure and activities in a forest environment [13].

Likewise, many empirical studies of which showed that a forest environment affects humans in both physical and mental health have been released. However, the previous studies focused on experiments about the crossover comparison of the groups through certain activities like meditation, walking, or recreation [14,15,16,17]. On the other hand, the experiments that attempted to analyze the factors for forest therapeutic environment are relatively insufficient [18,19]. Such indicators like light, wind, anion, or thermal environment can be factors for forest therapeutic environments, and the technologies to measure those indicators have been in progress. NVOC are either liquid or gaseous organic compounds that have boiling points below 100 °C and are easily evaporated into the atmosphere. Therefore, there have been some financial difficulties in the measurement and analysis of NVOC including analysis specimen and other technical problems such as different concentration values depending on the operators. Thus, this study attempted to develop a prediction model for NVOC concentration based on a microclimate environment in order to predict NVOC concentration more effectively. The study was also performed to scientifically suggest the potentiality of a forest as a healing space by providing real time information of NVOC concentration.

## 2. Materials and Methods

### 2.1. Study Site

**Figure 1:**
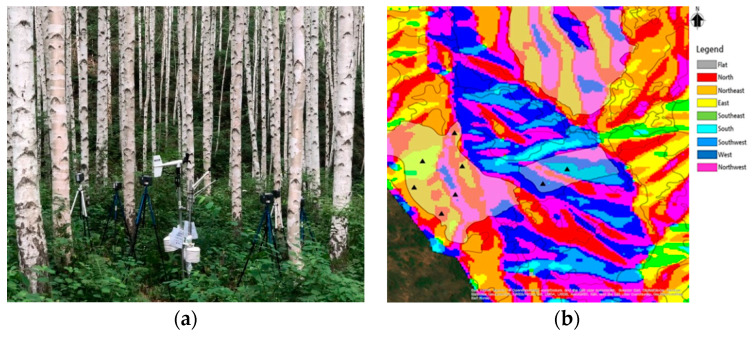
General current status of the research designated area. (**a**) Placing measurement devices on the site. (**b**) The classification map of site types.

The study site is a birch forest located in the wondae-ri *Betula platyphylla var. japonica* forest, a mountain Maebong (37°58′42″ N, 128°14′50″ E), Korea (Figure 1). The birch forest contains 700,000 trees planted in 138 ha. In order to study the geographic conditions of the subjects, we studied their aspect, altitude, and slope using GIS (Geographic Information System) and we used the quadrat method to study the vegetation.

The research object was located at an elevation of 690 m with an average slope grade greater than 15°, which is a mild slope. There was no state of erosion and the forest had high humidity, low wind exposure, and its aspect was southeast and northeast from the mountain valley. This forest tourism site opened in 2012 and is listed as one of the beautiful forests by the Korea Forest Service. The forest is popular with the locals in all seasons, particularly in winter for its snow-covered landscape. The wondae-ri birch forest is visited by over 230,000 visitors annually. This tree, known as the Japanese white birch, has been used in traditional treatments for various purposes such as pneumonia, nephritis, and chronic bronchitis [20,21,22].

**Figure 2:**
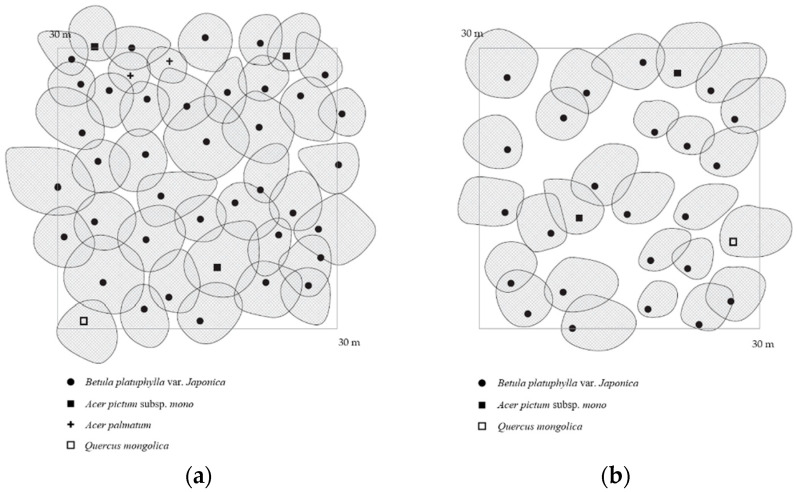
The crown projection of research stands: (**a**) crown density: 90%, (**b**) crown density: 75%.

A 30 m × 30 m quadrant was established at the NVOC measurement site, and plant species were divided into three categories: tree layer, subtree layer, and shrub layer to conduct a plant sociological survey (Figure 2). Records of the average tree height and crown projection charts of the tree layer were prepared. A log-wood survey of the tree species measuring ≥2 cm in diameter at breast height (DBH) in the study site was performed. The white birch forest in the research area covered with *B. platyphylla* was approximately 23 m high with a 50 cm diameter of breast height. Its sedimentary form is creep and forest soils are brown forest soils.

### 2.2. Measurement Factors

The study concentrated on methods for analyzing NVOC. Terpene compounds, a type of NVOC emitted by trees, are produced from multiple base units of isoprene, which contains five carbon and eight hydrogen atoms. Terpenoids, a common and large NVOC group, comprise hemiterpenes, monoterpenes, and sesquiterpenes. Emission inventories show that isoprene and monoterpenes are the most prominent compounds. These compounds are usually strong smelling, rarely water soluble and found in plants, animals, and microorganisms [23].

**Figure 3:**
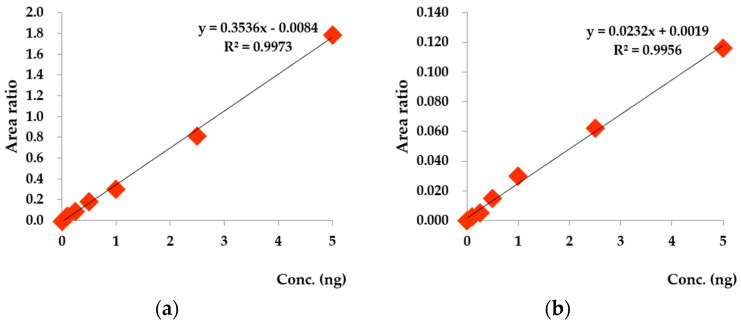
The calibration curve of measurement materials: (**a**) α-pinene calibration curve, (**b**) camphor calibration curve.

For selecting the standard substances for the analysis, literature research on both substances, ones that were analyzed by previous NVOC experiments [24,25,26,27,28], and the others that were medically verified for affecting the human body has been proceeded [29,30,31,32,33,34,35,36,37,38,39,40,41,42,43,44]. In the study, 36 species of monoterpenes and sesquiterpenes (99%, Sigma-Aldrich, St. Louis, MO, USA) including α-pinene, β-pinene, and camphene were selected for the analysis (Figure 3). There are a few tests to confirm the results of both the analysis device and the procedures. First, this study used 36 species of standard materials such as α-pinene and β-pinene to obtain the calibration curve. Using the calibration curve to calculate each element’s mass number and the square of its rate of diluting standard materials, it was found that the majority of the materials had a linearity greater than 0.997 (e.g., α-pinene (R^2^ = 0.997), β-pinene (R^2^ = 0.998), and d-limonene (R^2^ = 0.999)). The experiments using these materials also had a high reproducibility with respect to the coefficient of determination, which is suitable for the analysis.

### 2.3. Measurement Methods

#### 2.3.1. Natural Volatile Organic Compounds

The study sites were set up at 20 m intervals according to the geographical characteristics in the shape of a circle. Six pumps were placed at each point of the study site in consideration of the vegetation characteristics. The NVOC concentration detected from *B. platyphylla* increased over time from sunrise to sunset. The time was measured based on the Korea Meteorological Administration’s weather forecast time for sunrise (05:00), culmination (12:20), and sunset (19:30). The adsorption tube method was used to collect samples. Tubes (Markes, Sacramento, CA, USA) filled with Tenax TA (150 mg) were used for adsorption. The sample capture device was a mini pump (MP-∑30KN, Sibata, Japan) and the calibration was proceeded by a calculation of the adsorption error prior to the use of the flow meter. A total NVOC volume of 9 L was collected at a flow rate of 150 mL/min. The sampling equipment was installed on a tripod, 1.5 m from the ground, and the average value was calculated through duplicate sampling at every location. Disposable polyethylene gloves and antibacterial masks were used to prevent artificial errors when in contact with the tube during the installation. After sampling, the sample tubes were maintained at a temperature below 4 °C and analyzed within 48 h (Table 1).

The samples were subjected to qualitative and quantitative analyses using a gas chromatography-mass spectrometer (7890N-5975, Agilent, Santa Clara, CA, USA) with a thermal desorption system (GC-MSD, Gerstel TDS, Gerstel, Germany). The substances adsorbed by the adsorption tube were concentrated in a low-temperature cryofocusing device, which intakes high-purity helium gas at a velocity of 1 mL/min from a thermal desorption device. The device desorbed the gas for 3 min at 210 °C and maintained its temperature at −30 °C. The substances were then subjected to thermal desorption for 3 min at 220 °C, infused into a GC spectrometer, and detected using a MSD.

#### 2.3.2. Microclimate Environment

The direction and slope of the site were measured in terms of locational environment. A setup was designed to ensure that a portable multifunction meter (HOBO-U23 V2, Onset, Bourne, MA, USA) recorded the physical features of the site environment (temperature, humidity, dew point, globe temperature, air current, and wet-bulb globe temperature) at intervals of 5 min. Each of solar radiation sensors (S-LIB-M003, Onset, Bourne, MA, USA) and the photosynthetically active radiation sensor (S-LIA-M003, Onset, USA) was placed on the same spot and began monitoring throughout the entire experiment. Furthermore, a wind monitoring sensor (Wind monitorO5103-45, R.M.Y., Logan, UT, USA) was placed in consideration of the geological features in order to obtain the data of wind direction and velocity at the designated site. The meter was installed at a height of 1.5 m in equilibrium on a tripod approximately 5 m from a mini pump and digitalized measurement results were saved and then converted for the analysis. The results were analyzed using the HOBO-ware Pro program (Onset, USA). The data was saved 5 min before and after each measurement were excluded from the analysis to minimize measurement errors.

#### 2.3.3. Data Analysis

The data were analyzed for a total of 380 samples. Among them, 98 samples were excluded from the analysis (the 98 samples that were detected as high concentration by unspecific factors were considered as robustness weights). All statistical analyses were performed using Statistical Package for Social Science software, Version 23 (IBM Corp., SPSS Inc., Armonk, NY, USA). In this study, Pearson’s correlation coefficient was used as the main correlation analysis, as it is most commonly used to statistically measure the level of correlation among the variables. Among the regression analysis, the stepwise regression analysis method was used because it only contains the variables that are able to influence the dependent variable into the equation. In order to certify the multiple regression model’s significance, the VIF coefficient was used to check multicollinearity.

## 3. Results

### 3.1. Characteristics of NVOCs at Betula platyphylla Forest

**Figure 4:**
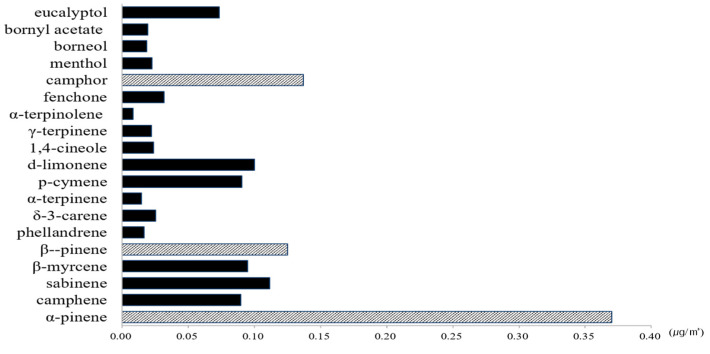
The characteristics of NVOCs at *Betula platyphylla* forest.

The comparison of the construction of NVOCs in the *Betula platyphylla* forest in the spring and summer seasons are shown in Figure 4 with 27% of α-pinene (0.37 μg/m^3^), followed by 10% of camphor (0.16 μg/m^3^) and 9% of β-pinene (0.13 μg/m^3^).

The other majority of NVOCs emitted sabiene (0.11 μg/m^3^), d-limonene (0.10 μg/m^3^), β-myrcene (0.10 μg/m^3^), p-cymene (0.09 μg/m^3^), camphene (0.09 μg/m^3^), eucalyptol (0.07 μg/m^3^), fenchone (0.03 μg/m^3^), δ-3-carene (0.03 μg/m^3^), 1,4-cineole (0.02 μg/m^3^), menthol (0.02 μg/m^3^), γ-terpinene (0.02 μg/m^3^), bornyl acetate (0.02 μg/m^3^), borneol (0.02 μg/m^3^), phellandrene (0.02 μg/m^3^), α-terpinene (0.012 μg/m^3^), and α-terpinolene (0.01 μg/m^3^).

### 3.2. The Intraday Variation of NVOC Concentration at the Betula platyphylla Forest

**Figure 5:**
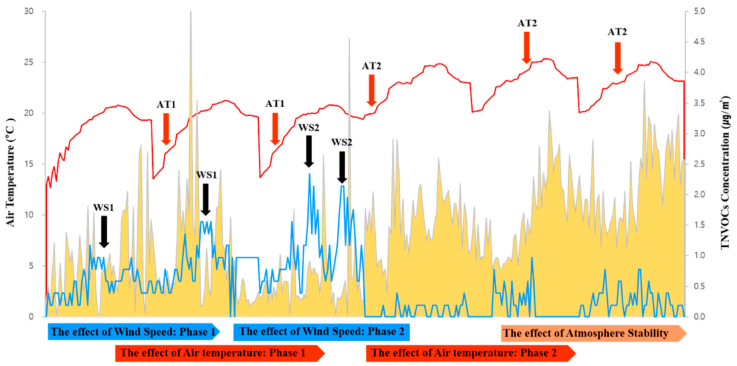
Concentration effect of NVOC by the influence of wind speed and air temperature.

To evaluate the changes in NVOC concentration during the day in the *B. platyphylla* forest, NVOCs were measured nine times at each point of the study site. Nineteen substances in total including α-pinene and β-pinene were detected in which α-pinene and camphor were detected at high concentrations during the sunset. Thus, based on a thorough investigation of other substances that were detected at low concentration, it was confirmed that sabinene, α-terpinene, γ-terpinene, α-terpinolene, etc. were detected at high concentrations during culmination. Most of the cases were influenced by wind velocity, but it was confirmed that some of the substances experienced the change in concentration by the photo environment, rather than by the influence of wind velocity and temperature; for example, VOC substances had a half-life by the optical reaction (Figure 5).

### 3.3. Relationship between NVOC and Microclimate at the Betula platyphylla Forest

To maintain the accuracy of the measured values, data taken from the first 5 min of each measurement was excluded. As a result, a negative linear relationship was confirmed between NVOCs and wind velocity where wind velocity decreased with increasing concentration of NVOCs. A positive linear relationship was confirmed with the temperature and humidity, showing that the temperature and humidity increased with increasing concentration of NVOCs. Microclimates and materials showing abnormal values in correlation analysis were excluded, and the excluded factors were analyzed, followed by reanalysis (Table 2).

### 3.4. Prediction Model for NVOC Concentration in Betula Platyphylla Forest

This study conducted a multiple regression analysis to create the NVOC concentration prediction model for changes in the microclimate environment (Table 3). To verify the independence of the residuals, the Durbin–Watson values were evaluated; a value of 1.990 was obtained, which showed no autocorrelation. The variance inflation factor (VIF) in this study was 1.479–3.016 and it indicated no multicollinearity issues. The results of the study site analysis presented a significant regression model with an F value of 83.296. The R^2^ was as high as 60.1%, confirming that the regression model of the concentration prediction of NVOC in *B. platyphylla* forest had significant explanatory power. Analysis of variance (ANOVA) showed a significantly high P value of 0.000 and the explanatory power of the regression model equation was high.

For temperature, the value of B was 0.096 and the test statistic was confirmed to have a significant effect with a t value of 8.914 and significance probability of 0.000. Due to the fact that the standardized beta value was 0.412, the concentration of NVOC increased by 0.412 (41.2%) when the temperature increased by 1 °C. In further analysis, the value of B was analyzed as 0.008 and the test statistic was confirmed to have a significant effect with a t value of 4.492 and significant probability of P of 0.000. Due to the standardized beta value of 0.296, the concentration of NVOC increased by 0.296 (29.6%) when humidity increased by 1%. For wind speed, the value of B was −0.261 and the test statistic showed that the t value was −3.164 and significance probability was 0.002, which was significant. As the standardized beta value was −0.190, the concentration of NVOC decreased by −0.190 (19.0%) when wind speed increased by 1%. In order to predict the concentration of NVOC in the *B. platyphylla* forest, independent variables influencing the concentration were selected from the microclimate by correlation analysis, followed by multiple regression analysis to create the following equation of the Model of NVOC concentrations from *B. platyphylla* forest: MNF ver 1.0 (Theorem (1)).

MNF (ver 1.0) = −1.204 + 0.096 × (Temp) + 0.008 × (Hum) −0.261 × (Vel) (1)

## 4. Discussion

We aimed to establish a prediction model for the concentration of NVOCs by comparing the correlation between microclimate environment within a forest and NVOC concentration. The changes in NVOC concentration depend on the interaction of inner factors of which genetic and biochemical factors [45,46,47,48], and outer factors: biological factors of animals, plants, microorganisms, and non-biological factors including temperature, sunlight, relative humidity, and wind velocity [49,50,51,52,53,54]. The NVOC concentration and its emitted mechanism are correlated by various factors, which may lead to inadequate outcomes with accurate mechanism. Additionally, previous studies on NVOCs have reported that the difference in influence is great depending on countries, regions, and climate. Generally, when it comes to comparing seasonal concentrations, the concentration in summer was confirmed to comprise the biggest proportion [49,55,56]. After researching the characters of the substances in each season, some particular substances tended to become lower when the temperature was raised. The studies on the influence based on humidity, however, are relatively insufficient. Most of the previous studies have indicated that the concentration increases in proportion to humidity. It has also been reported that higher concentration is detected in times of drought. After a comparison to the previous studies, it was decided that the microenvironment is a factor of change mechanism in concentration [55,56]. Thus, this research aimed to investigate the relationship between NVOC concentration occurring in a *B. platyphylla* forest (a broad-leave tree representing a northern-temperate forest) and microclimate, one of the mechanism factors. This study was also different from some of the previous studies due to its focus on analyzing the NVOC concentration by developing the prediction model and establishing a correlation between a microclimate environment and NVOC concentration. The previous studies mainly proceeded experiments of collecting NVOC from above the canopy, or changing particular factors by closing a leaf in a Tedlar bag. There are many studies about the relationship between effects to emission and NVOC concentration within artificially given conditions, but not between NVOC concentration and the microclimate environment in the forest. NVOC concentration is one of the evaluation criteria to select a ‘healing forest’, but there are financial difficulties like collecting specimens for measuring and analyzing NVOCs. Furthermore, technical problems like observing different concentration rates depend on how the operators could occur. Additionally, it is impossible to check the concentration in the whole study area at the same time due to the limits of measuring devices, and the fact that NVOCs change in their concentration depending on the condition of the microclimate environment within the forest. Therefore, this study attempted to develop a prediction model for the NVOC concentration by the microclimate environment in order to predict the analysis of NVOCs more efficiently.

After proceeding the study based on the study purpose above-mentioned, the characteristics of the relationship between microclimate and NVOC concentration that was emitted from a birch forest in spring and summer were deduced. Intra-day measurement of changes in NVOC concentrations found that concentrations were higher after sunset, when the boundary layer of the atmosphere contracted, stabilized, and the photo-environment changed. This is consistent with the result showing that the atmosphere is stable and not mixed well at night time compared to during the day time when it is perished by rapid reactions with oxidants such as O_3_ in the atmosphere, although there may be some differences in the concentration changes between the atmosphere in forests and the air above the canopy [57,58]. In addition, NVOC concentrations increased when the photo-environment changed and decreased when the wind velocity changed. Among the detected substances, sabinene, α-terpinene, γ-terpinene, and α-terpinolene were highly concentrated during culmination, and thus showed lower reactivity with the photo-environment than other substances. The correlation between NVOC concentration and microclimate demonstrated that higher temperature and humidity were associated with a higher NVOC concentration whereas higher wind speed resulted in a lower concentration. The results of this study are similar with those in coniferous forests in previous studies, but there were some differences in substances, which leads to the conclusion that there are differences in the composition of substances [59,60,61].

However, this study has some experimental limits such as the model is only able to predict the concentration in certain microclimate conditions. First, pure forests are more widespread than mixed forests are in South Korea. Additionally, uneven-aged forests have a more complicated composition than even-aged forests, so new concentration models of NVOCs by location or position other than by species of trees needs to be developed. Second, the mechanism factors of occurrence interact with each other. Thus, in addition to the microclimate environment that was utilized in this study, the correlation between the various internal and external factors like biomass, leaf area, flowering, PAR, soil, stand age, density of standing trees, and evapotranspiration needed to be checked. Third, seasonal NVOCs concentrations for each forest floor needed to be monitored because this study monitored other research sites only for spring and summer. Fourth, the research related to NVOCs that national and academic institutes have undertaken are mostly based on the chemical natures of the substance. However, NVOC substances have an effective half-life in atmosphere, and a kind of substance exists as various isomers. Additionally, the substance for analysis based on the purpose it is needs to be selected because NVOCs can be found not only in trees, but also in herbs. Finally, the study of NVOCs can result in various conclusions, depending on the operators. The measuring processes for NVOC concentration in South Korea are mostly on the basis of indoor air quality processes without specific guidelines. Therefore, it is necessary to set up a standard process that is scientifically and technologically proven through the feedback from experts in air or forest analysis.

In facing the transition to a welfare state and an increase in social demand with regard to welfare, the forest sector should take a role that aims for the expansion and advancement of forest therapy. In fact, the studies for measuring the forest effects on the human body both physiologically and psychologically are increasing; however, so too is the necessity of fundamental research along with the awareness of reconsidering healing environments. The fundamental experimental research on directly proving the therapeutic factors within forests is still insufficient. To revolve such shortages, fundamental scientific research development on forest therapy environmental factors should be conducted. This study is expected to be able to help the public understand a new side of the forest environment and predict NVOC concentrations more efficiently in order to select or promote therapeutic forests in the future, and provide fundamental data for the forest sector’s health-medical value. Furthermore, this study explored what influence organic chemicals in the atmosphere changed inside the forests that were known for their therapeutic effects.

## 5. Conclusions

To achieve sustainable development of white birch forests, this study was conducted to identify the potential of these forests as healing spaces with forest value. Additionally, the significance of a northern temperate forest environment due to climate change was analyzed. To complement the financial and technical issues of existing NVOC measurement techniques, the study conducted a total of 380 samples and analysis from a *B. platyphylla* forest. Analysis of the NVOCs emitted at the study site showed that 19 types of substances were detected. The comparison of the construction of NVOCs in the *B. platyphylla* forest showed 27% of α-pinene (0.37 μg/m^3^), followed by 10% of camphor (0.16 μg/m^3^), and 9% of β-pinene (0.13 μg/m^3^). In this study, a prediction model for the NVOC concentration of *B. platyphylla* was proposed considering the concentration changes related to the microclimate, and thee regression analysis showed an explanatory power as high as 60.1%. However, with the lack of validation samples and high analysis costs, it is necessary to obtain samples for comparative validation in future studies and the model should be supplemented after considering various physical factors. This study developed a model to effectively predict NVOC concentrations. Forests are spaces that act as a buffer between nature and humans. Unlike recreational forests that are standardized, therapeutic forests should be a multilateral space that can play multi-roles depending on the change of functional requirements. With the results of this study, it is hoped that the fundamental value of forests is reconsidered. Furthermore, it is expected that the prediction model for NVOC concentration that this study proposes will be more connected to the data from the forest meteorology net of South Korea, so that it can provide real time information of NVOC concentration in the therapeutic forests in the country.

## Figures and Tables

**Table 1:** The conditions for the operating parameters for NVOC.

Parameters	Conditions				
Column	HP-INNOWAX (60 m × 0.25 mmL Dx 0.25 μm, film thickness)				
Carrier gas flow	He at 1 mL/min				
Injection mode	Pulsed Splitless				
Injection port temp.	210 °C				
Transfer line temp.	210 °C				
Oven temp. program	initial		Rate	final	
	40 °C	3 min	8 °C/min	220 °C	3 min
Post run	220 °C, 5 min				

NVOC: Natural volatile organic compounds.

**Table 2:** Correlation between NVOCs and the *Betula platyphylla* forest microclimate.

Variables		TNVOC	Temp. ^1^	Hum. ^2^	Vel. ^3^
TNVOC	correlation	1	0.649 **	0.681 **	−0.567 **
	Sig.		0.000	0.000	0.000
	N	282	282	282	282

^1^ Temperature, ^2^ Humidity, ^3^ Wind Velocity (Pearson’s correlation was used ** *p* < 0.01).

**Table 3:** Result of multiple regression analysis for *Betula platyphylla* forest.

Variables	SE	Beta	t	VIF	R^2^	F	D-W
Constant	0.219		−5.501		0.601	83.296 (0.000)	1.990
Temp. ^1^	0.011	0.412	8.914	1.479			
Hum. ^2^	0.002	0.296	4.492	3.016			
Vel. ^3^	0.082	−0.190	−3.164	2.492			

^1^ Temperature, ^2^ Humidity, ^3^ Wind Velocity.
